# Age, but not anthelmintic treatment, is associated with urinary neopterin levels in semi-free ranging Barbary macaques

**DOI:** 10.1038/srep41973

**Published:** 2017-02-03

**Authors:** Nadine Müller, Michael Heistermann, Christina Strube, Oliver Schülke, Julia Ostner

**Affiliations:** 1 Department of Behavioral Ecology, Johann-Friedrich-Blumenbach Institute for Zoology and Anthropology, Georg August University Göttingen, Göttingen, Germany; 2 Endocrinology Laboratory, German Primate Centre, Leibniz Institute for Primate Research, Göttingen, Germany; 3 Institute for Parasitology, Centre for Infection Medicine, University of Veterinary Medicine Hannover, Hannover, Germany; 4 Research Group Primate Social Evolution, German Primate Centre, Leibniz Institute for Primate Research, Göttingen, Germany

## Abstract

Studying host parasite interactions and their implications for evolution and ecology recently received increasing attention, particularly with regard to host physiology and immunity. Here we assess variation of urinary neopterin (uNEO), a marker of cellular immune activation and iummunosenescence, in response to age and anthelmintic treatment in semi-free ranging Barbary macaques (*Macaca sylvanus*). Urinary NEO levels were measured via enzyme-immunoassay from 179 urine samples of 43 individuals between 5–29 years of age. Efficiency of treatment was assessed by Mc Master flotation on repeated faecal samples, including 18 untreated individuals as control group. We used linear mixed models with age and parasite status as main effects, controlling for sex and physical condition, assessed through urinary C-Peptide-levels, with social group and ID as random factors. Urinary NEO levels significantly increased with age, suggesting that changes in aging Barbary macaque immune responses are consistent with immunosenescence described in human and nonhuman primates and can be detected via uNEO measurements. Anthelmintic treatment, however, had no influence on uNEO levels, potentially due to quick reinfections or attenuated immune responses in repeated infections. We conclude that uNEO is a potential non-invasive marker for immune function and particularly immunosenescence in wildlife.

Infectious diseases have crucial impacts on the ecology and evolution of species, influencing numerous aspects of host life history, survival and fitness^1^,^2^ and are considered a major cost of group living^3^. Infections have been studied intensely in humans^4^,^5^ and model species^6^,^7^, and recently investigations of wildlife diseases and immune response have received increasing attention^8^. Recent advances in the field of studying senescence in wildlife^9^ demonstrate that senescence occurs in wild animals and consistently changes their physiology and immune response^10^,^11^. To understand the importance of age related changes and host-pathogen interactions for evolution, studying diseases and immune responses in natural systems has become crucially important. Wildlife health monitoring has focused on different aspects of an animal’s physiology that can be measured non-invasively, e.g. glucocorticoid excretion as a measure of physiological stress response^12^,^13^, testosterone excretion due to its supposed, though debated, immunosuppressive effect^14^,^15^, monitoring physical condition using visual criteria^16^,^17^, wound healing^18^, and measurements of C-Peptide of insulin^19^,^20^ and thyroid hormones^21^,^22^ as markers for energetic status. Studies of age related changes currently rely mainly on invasive sampling^10^,^11^, however, capturing and handling animals can be difficult or impossible and raise particular ethical concerns for endangered species or species sensitive to disturbance. Problems associated with invasive sampling make comparative work on health status challenging, so the development of non-invasively obtained measures of health- and age-related parameters are critical to this area of inquiry.

Information about immune responses of individuals with regard to certain pathogens and aging are necessary to understand the impact of infection on host fitness, as immune responses themselves are energetically costly or lead to energy allocation away from reproduction^23^. Using blood samples is highly efficient when studying immune responses on the molecular level and have generated major insights into host-parasite interactions^24^ and the effects of intrinsic and extrinsic factors on immune responses and reactivity^10^,^25^. One current challenge in the fields of wildlife senescence and eco-immunology is the validation and establishment of suitable markers to monitor immune responses non-invasively (e.g. using faecal and urine samples) in order to better understand host parasite interactions and their implications for fitness in a wider range of systems.

Recently, advances have been made validating assays to measure markers of immune response in faecal and urine samples from nonhuman primates^26^. One particularly promising marker is neopterin (NEO)^26^,^27^, which is released from macrophages, monocytes and dendritic cells in response to interferon gamma (INFγ) stimulation^28^ and induces T-helper 1 cell activation in the immune response against intracellular pathogens such as viruses and bacteria^28^,^29^. Being also elevated in a variety of inflammatory^30^ and non-infectious diseases^28^,^31^ and even under acute stress^32^, NEO may serve as a non-disease-specific marker of inflammation and Th1 mediated immune responses and is widely used as a diagnostic marker for disease severity and prognosis in humans in a variety of diseases^28^. NEO is cleared unchanged via the kidneys^33^ and serum and urinary NEO levels are strongly correlated in both human^34^ and nonhuman primates^26^,^35^. Supporting limited findings of an earlier study^35^, uNEO levels increased more than 10-fold following infection with Simian immunodeficiency virus (SIV)^26^, indicating the usefulness of uNEO level changes as a non-invasive marker of viral infection in nonhuman primates.

Apart from being a marker of acute or chronic disease associated with a Th1 immune response^28^,^29^, NEO is a marker intensely studied with respect to age-related changes in the immune system. There are consistent changes in both, the innate and adaptive immune system, occurring with older age: decreased cellular immune response^36^ and efficiency of vaccinations^37^,^38^, changes in T-cell phenotypes with an increase in differentiated and simultaneous decrease in naïve T-cells^39^,^40^, alterations in the innate immune system, especially changed monocyte phenotypes^41^ as well as reduced natural killer cell function^40^,^42^, and chronic low levels of inflammation^40^,^43^. Elevated NEO levels in aged individuals have not only been reported in numerous studies^44^,^45^, but also correlate with several hallmark characteristics of the aged immune system in humans, amongst others altered T-cell phenotypes and changes in monocytes^41^,^45^, indicating NEO as a marker of age related immune function changes.

Given the growing importance of understanding healthy aging and the processes leading to frailty and morbidity with older age in aging human societies^46^, nonhuman primates have become an increasingly important model system to study immunosenescence^47^,^48^, a term that summarizes the changes in the immune system of aging individuals^49^. To date, most studies on immunosenescence in nonhuman primates report levels of IFNγ rather than NEO, which in most studies are elevated in older individuals^50^,^51^. Since IFNγ and NEO are closely linked functionally^28^, similar patterns are expected for NEO in aging nonhuman primates. This is supported by a recent study that reported a significant positive correlation between serum NEO levels and age in healthy macaques^26^.

We aimed at advancing the biological validation of uNEO in nonhuman primates with regard to aging by using a cross-sectional design of young adult to aged free ranging Barbary macaques (*Macaca sylvanus*). Additionally, we aimed at assessing whether uNEO levels decrease in response to gastrointestinal (GI-) nematode infection, a group of pathogens that have been widely studied in nonhuman primates^12^,^52^ and other taxa^53^,^54^. We capitalized on routine six-monthly anthelmintic treatment and assessed the impact of parasite clearance on uNEO levels in a cross-sectional design. Since both arms of the immune system, Th1 and Th2 are mutually inhibitory^55^, high levels of NEO not only represent an efficient Th1, but also an inhibited Th2 response. As immune responses against GI-nematodes generally share a strong antibody mediated and anti-inflammatory Th2-type response^56^, uNEO can potentially function as not only a direct marker of intracellular, but also an indirect marker of parasite infections. A similar relationship between GI parasite infection and Th1 responses was demonstrated in wild African buffalos (*Syncerus caffer*), where individuals with lower Th1 activity were less vulnerable to GI-nematode infections^24^ and Th1 responses increased as a result of experimental parasite clearance. Since direct energetic costs of immune responses, including parasite defences, have been shown in several taxa^57^,^58^, and clinical studies on humans have linked NEO levels to markers of energy status^59^,^60^, we integrated a non-invasive measure of energy balance, urinary C-Peptides (uCP)^20^,^61^, into our analyses to control for possible confounding effects of physical condition on uNEO values. We investigated the relationship between aging, GI parasite infection and uNEO levels in semi-free ranging Barbary macaques at *Affenberg Salem* in a population with a high proportion of aged individuals older than 20 years (19% of the study population). We specifically predicted increasing uNEO levels with increasing individual age in adulthood, representing changes in the immune system consistent with immunosenescence. Individuals older than 20 years were expected to have the highest uNEO levels. Additionally, we used routine anthelmintic treatment of the population to investigate the impact of GI parasite clearance on uNEO. Based on the findings of Ezenwa *et al*.^24^,^54^, we predicted uNEO levels to be increased after treatment due to lack of immunomodulation by parasites.

## Materials and Methods

### Study site and urine sample collection

We studied two out of three freely interacting social groups of Barbary macaques in a 20 ha forested enclosure at *Affenberg Salem* in Germany. Macaques were provided with fruit, vegetables, and grains daily in the morning and had *ad libitum* access to monkey chow and water^62^. The two study groups had roughly similar age-sex compositions (group C: 20 adult females, 16 adult males, 11 immature females and 12 immature males; group H: 23 adult females, 18 adult males, 3 immature females, 8 immature males). All individuals were identifiable by physical features such as birth marks, scars, stiff fingers or stature. Urine samples from all adult individuals were collected repeatedly from individually recognized individuals six weeks prior to anthelmintic treatment to allow for collection of baseline pre-treatment data for the study population. Sampling was continued until four weeks post-treatment (group C: 2^nd^ of June through 26^th^ of August 2014, group H: 6^th^ of June through the 9^th^ of September 2015). The four weeks window was chosen to allow for collection of several samples per individual while being able to assess the effect of reduced parasite burden. Since prepatant phases of strongyle nematodes present in the population are approximately two to three weeks^63^ and most individuals started shedding eggs six weeks after treatment or later (unpublished data), we are confident this was actually the case. Only samples of 43 adult individuals (7 of group C, 36 of group H) with at least 1 sample pre- and post-treatment were analysed (179 samples, 4.2 ± 1.3/individual, pre-treatment: 93 samples, 2.2 ± 0.8/individual, post-treatment: 86 samples, 2.0 ± 0.9/individual). The 22 sampled females were 5–29 years of age and the 21 males 6–27 years of age (Table 1). Samples were taken non-invasively using one of two methods: Urine was caught on clean plastic sheets when individuals were urinating from elevated positions (higher than 2 m) and subsequently transferred into 2 ml polypropylene cups with disposable Pasteur pipettes. We aimed at collecting samples from every individual, but had difficulties to obtain samples in 2014 as Barbary macaques are largely terrestrial and samples could not be collected when individuals urinated close to the ground. In 2015 we increased our sampling effort in order to obtain samples from more individuals. In addition to collecting urine by catching it on plastic sheets we collected urine from the ground, rocks or leaves using swabs (Salivette^®^ Cortisol, Sarstedt, Nürmbrecht, Germany) when individuals urinated close to or on the ground. The latter method has recently been validated for NEO measurements in macaque and human urine^64^ and allowed for a much higher sampling success. Samples contaminated with faeces were discarded as faecal contamination alters levels of uCP in macaque urine samples^65^. Following collection, both salivettes and polypropylene cups were stored on ice in a thermos flask until transferring them to −20 °C for long-term storage within 12 h of collection^27^. Urine was recovered from the salivettes by centrifugation using a manually operated centrifuge^64^ and was transferred to 2 ml polypropylene cups with disposable Pasteur pipettes prior to freezing. All urine samples were transported to the Endocrinology Laboratory of the German Primate Centre (Göttingen) on dry ice and stored frozen at −20 °C until analysis.

### Anthelmintic treatment, parasite analyses and treatment efficacy

Anthelmintic treatment was performed on the 5^th^ of August 2014 and 17^th^ of August 2015 as a veterinary routine measure. Individuals were fed food items containing ivermectin, a broad spectrum anthelmintic compound belonging to the macrocyclic lactone drug class, at approximately 0.4 mg/kg bodyweight, by park staff in close collaboration with the veterinarian responsible for the population. In 2015, half of the individuals of group H remained untreated as a control group (n = 18), which was matched for sex, age, immigration status (males) and matriline (females) to the treatment group (nine females, 5–29 years, nine males, 6–27 years of age).

Since we aimed at investigating the impact of age and parasite status on uNEO levels, confirming the efficacy of anthelmintic treatment was one prerequisite for the analyses. To do so we collected faecal samples for every individual approximately once per week for parasite analyses (pre-treatment: n = 294, 6.7 ± 0.9/individual, post-treatment n = 292, 3.8 ± 1.4/individual). Samples were collected as soon as possible (without disturbing the animal) after defecation noting animal ID, date, time, and observer, placed in 20 ml plastic tubes, stored on ice until being fixed with 10% formalin within 12 hours and later transported to the Institute for Parasitology of the University of Veterinary Medicine Hannover, Germany. Presence of nematode eggs was assessed using McMaster flotation, a quantitative parasitological method, after wash-out of formalin from the samples by transferring ca. 6 g to a 15 ml centrifuge tube, spinning at 2000 rpm (930 g) for 10 min, washing once with water, spinning at 2000 rpm (930 g) for 10 min and discarding the supernatant^66^. For McMaster flotation, 4 g faecal matter was weighed, homogenized in saturated NaCl as flotation solution, poured through a tea strainer (mesh size 1 mm) to remove faecal particles and the sample was filled up to 60 ml with saturated NaCl. For samples with less faecal matter available, 15 ml flotation solution were used per gram and the weight of faeces used was recorded. Faecal egg counts were performed by using McMaster chambers and scanning 4 counting fields with 100- and 400-fold magnification (detection sensitivity 25 eggs/gram faeces), recording presence of parasite egg morphotypes.

Coproscopical parasite analysis revealed three nematode morphotypes, for which effectiveness of treatment differed: for strongyle nematodes, which had a pre-treatment prevalence of ~98% (n = 42 individuals), all treated individuals stopped shedding eggs within two days of treatment and stayed coproscopically negative for at least three weeks. One individual excreted eggs again within three weeks and three individuals within four weeks. All other treated individuals remained coproscopically negative for at least four weeks after treatment. As strongyle eggs are difficult to differentiate by morphological characteristics, we summarized several potential genera under this morphotype, e.g. *Oesophagostomum* spp. and hookworms (*Ancylostoma* spp. and *Nercator* spp.). Preliminary results from larval cultures suggest that the majority of strongyles represent *Oesophagostomum* spp., whereas only few samples contained other larval morphotypes (unpublished data). Given the short prepatent periods of the possible parasite species^63^, this result indicates parasite clearance with rapid reinfection in few individuals. For the two other morphotypes, *Capillaria* spp. and *Trichuris* spp., prevalences were lower (*Capillaria* spp.: 37.2%, n = 16 individuals; *Trichuris* spp.: 6.9%, n = 3 individuals), but anthelmintic treatment was not efficient for all treated individuals as indicated by continued egg shedding (two study individuals and seven additional treated individuals not included in the study for lack of urine samples).

### uNEO and uCP measurements

We assessed uNEO concentrations measuring duplicates for each sample using a commercial Neopterin ELISA Kit (Art. No. RE59321, IBL International GmbH, Hamburg, Germany) previously validated for the use in macaques^26^,^27^. Prior to assay, urine samples were diluted 1:25 to 1:250 with assay buffer and 20 μl of the diluted urine was subsequently assayed using the manufacturer provided protocol. In this study, uNEO concentrations are provided as ng/ml by transforming the respective nmol/l values according to the manufacturer’s protocol. Sensitivity of the assay was 0.34 ng/ml. Intraassay coefficients of variation (CV), determined by repeated measurement of high and low value quality controls in each assay, were 5.4% (high; n = 18) and 8.1% (low; n = 18), respectively, while interassay CVs were 6.1% (high; n = 5) and 10.5% (low; n = 5).

In addition to uNEO levels, we measured uCP concentrations as a control factor to account for individual differences in physical condition and energy balance^19^,^20^ as these might influence the ability of individuals to mount effective immune responses^58^,^67^ and influence uNEO levels^57^,^59^,^60^. We analysed uCP measuring duplicates of each sample using a commercial C-Peptide ELISA Kit (Art. No. RE 53011, IBL International GmbH, Hamburg, Germany) previously validated for the use in macaques^20^,^65^. Prior to assay, urine samples were diluted between 1:2 and 1:20 with IBL sample diluent (Art. No. RE 53017) and 100 μl of the diluted urine was then assayed according to the manufacturer’s instructions. Assay sensitivity was 0.064 ng/ml. Intraassay CVs calculated from the measurement of high- and low-value quality controls (pooled human urine) were 5.2% (high; n = 18) and 8.8% (low; n = 18), respectively, while values for interassay CVs were 7.0% (high; n = 5) and 14.2% (low; n = 5).

Due to the expected sex differences in muscle mass and consequently creatinine excretion^68^, we did not use creatinine but specific gravity (SG) to adjust for differences in urine volume and concentration^68^. We measured SG of each sample using a digital hand-held refractometer (PAL-10S; Atago Inc., Bellevue, USA) and calculated uNEO levels and uCP levels corrected for SG by using the following formula^69^: 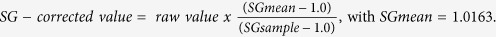


### Statistical analysis

We assessed the effect of age and anthelmintic treatment on uNEO levels using linear mixed models in R (version 3.5^70^) with the package lmerTest, setting alpha levels to 0.05. After exclusion of two extremely low uNEO values that repeatedly led to extremely high residuals and prohibited log-transformation to achieve normality, both uNEO as response and uCP as predictor were log-transformed to achieve normal distribution. Rather than z-transforming age, age was rescaled by subtracting the mean age (14 years) value from individuals’ values to centre the model around the mean age rather than the not meaningful value of age = 0 for the y-intercept. This was done to keep the original data structure and allow interpretation of the effect size.

Since *Trichuris* always occurred in individuals positive also for *Capillaria* both morphotypes were summarized under the factor “*Cap”* for statistical analysis. Based on the differences in anthelmintic treatment efficacy and the different outcomes for *Cap* negative (GI parasite free) vs. *Cap* positive (GI parasites still present), we modelled the predictors representing parasite treatment using a three-way interaction between experimental group (control vs. treatment), phase (pre- vs. post-treatment) and *Cap* (positive vs. negative) as main factor in the original model (Model 1), expecting smaller or no changes in uNEO levels in *Cap* positive vs. *Cap* negative treated individuals. The model contained the three-way interaction and age as main factors. As NEO has been shown to increase in elderly humans only rather than linearly across adulthood, we tested using a model with age as a quadratic term to account for the nonlinearity, however, the quadratic term was neither significant (estimate age^2^ = −0.001, t-value = −0.498, p = 0.621) nor improving the model fit (model comparison using a likelihood ratio test with the R function anova, setting the argument test to “Chisq”, Chisq = 0.390, p = 0.533), so we chose a linear fit for age. We included sex as a control variable, because rhesus macaque females showed higher levels than males in laboratory studies, though this effect was not significant^26^. The two-way interactions of *Cap*, experimental group and phase were included, albeit being not interpretable given the three-way interaction, as required for model validity. The single terms *Cap*, experimental group, phase as well as sex and uCP were included as control factors, social group and animal ID as random factors.

Since the three-way interaction was not significant we tested if its inclusion significantly improved the model fit as a measure of model selection. Model 1 was not significantly better fitted to explain uNEO level variability than the identical model including only the two-way interaction between experimental group and phase and the single terms experimental group, phase and *Cap* (Chisq = 5.369, p = 0.147). Consequently, we used the less complex model for interpretation of the results (Model 2), but report results of Model 1 for reasons of transparency.

For both models, various model diagnostics were employed to confirm model validity (visual inspection of distribution of residuals, qqplots, residuals plotted against fitted values, assessing model stability using the function “glmm stability” written by Roger Mundry (MPI Evolutionary Anthropology, Leipzig), assessing leverage and dfbetas for single samples and levels of the random factor ID using the package “influence. ME” and variance inflation factors using the package “car”), none of which suggested violation of model assumptions for either model. To assess the significance of the full models we compared them to reduced models excluding the age-term and the interaction-term between experimental group and phase while retaining all other predictors using a likelihood ratio test with the R function anova, setting the argument test to “Chisq”.

### Ethical statement

This work followed the Animal Behaviour Society’s guidelines for the treatment of animals in behavioural research and teaching, and adhered to standards as defined by the European Union Council Directive 2010/63/EU on the protection of animals used for scientific purposes. Anthelmintic treatment was performed as part of the routine procedures of *Affenberg Salem* as defined by the European Union Council Directive 1999/22/EC and authorized by the Veterinary Office of the district office of county Lake Constance. The study was approved by the Animal Welfare Body of the German Primate Center (No. E9-16).

## Results

### Influence of age on uNEO in semi-free ranging Barbary macaques

**Figure 1:**
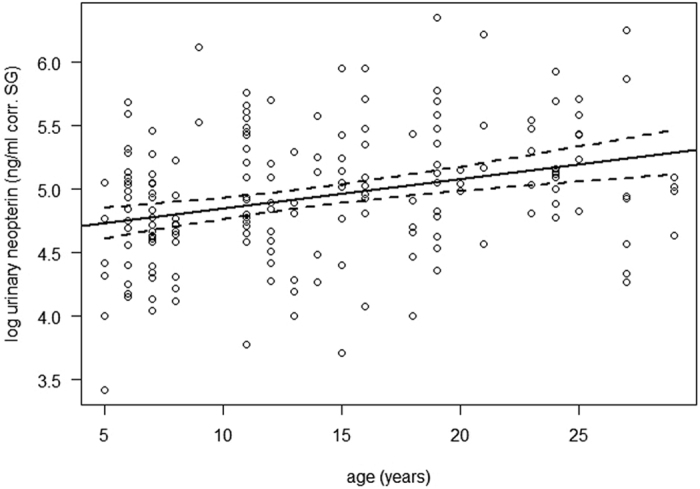
Effect of age on uNEO levels in 43 adult Barbary macaques. Regression line was fitted according to the estimates of Model 2 (n = 179 samples), broken lines depict 95% confidence intervals. Log-transformed uNEO-values have been plotted against the original age values to allow easier interpretation instead of age scaled to mean age used in the model (which changes the y-intercept, but neither estimates nor interpretation of the model).

This study was the first to measure uNEO in Barbary macaques. Urinary NEO levels varied between 30.7 and 572.7 ng/ml corr. SG, mean ± sd = 160.45 ± 90.50 ng/ml corr. SG (Table 2) and did not differ between the sexes in either of our models (Model 1: estimate ± se = 0.11 ± 0.10, t-value = 1.08, p = 0.29, Model 2: estimate ± se = 0.08 ± 0.10, t-value = 0.83, p = 0.41, see Table 3, Table 4). In accordance with our predictions, age was a significant predictor of uNEO levels, with older individuals showing higher uNEO levels (Model 2: estimate ± se = 0.02 ± 0.01, t-value = 2.45, p = 0.019). The size of the effect was rather small, with individuals increasing ~4% of the mean log(uNEO) in ten years (Fig. 1). However, the age effect was highly stable as indicated by the narrow confidence intervals (2.5%: 0.004, 97.5%: 0.034, see Table 4), and effect sizes are difficult to interpret due to the log-transformation of uNEO levels. Comparing the mean untransformed values between young adult (5–7 years) and aged individuals (>20 years), the difference translates into a 60% increase in uNEO levels from young to aged individuals (Table 2).

### Influence of anthelmintic treatment on uNEO levels

**Figure 2:**
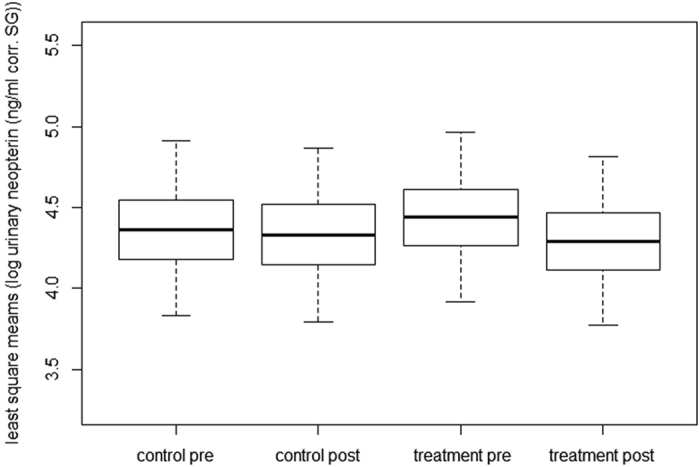
Effect of anthelmintic treatment on uNEO levels in 43 adult Barbary macaques. Depicted are least square means ± se and 95% confidence intervals of least square means for Model 2 for control and treatment group pre and post anthelmintic treatment (n = 179 samples: n control pre = 40 samples, n control post = 41, n treatment pre = 53, n treatment post = 45), visualizing the modelled interaction between experimental group and phase.

Including the three-way interaction of Model 1 changed the estimates, but not the directions of the effects (Table 3, Table 4) and did not improve the model fit, thus only Model 2 is discussed here. For Model 2, the full model was significantly better at explaining uNEO variance than a reduced model excluding age and the interaction-term between experimental group and phase (Chisq = 12.18, p = 0.032). Neither of the control factors (sex, *Cap*, uCP levels, single terms of experimental group and phase) had a significant effect on uNEO levels (Table 4). Contrary to predictions, we found no effect of treatment (interaction between experimental group and phase) on uNEO levels (Model 2: estimate ± se = 0.11 ± 0.13, t-value = 0.81, p = 0.42, Table 4), indicating that clearance of strongyle nematodes did not lead to significant changes in uNEO levels (Fig. 2).

## Discussion

In the present study, we aimed at investigating the effect of aging on uNEO in semi-free ranging Barbary macaques. Capitalizing on routine anthelmintic treatment, we assessed the potential impact of GI-nematode infection and age on uNEO levels. Anthelmintic treatment was successful for strongyle nematodes, whereas *Capillaria* and *Trichuris* infections prevailed in some individuals, which was taken into account when analysing the effect of treatment on uNEO levels. We ran linear mixed models to assess the impact of age and anthelmintic treatment on uNEO levels and found that only age showed a significant positive effect on uNEO levels, with aged individuals showing significantly higher levels than younger ones.

In both humans and the macaques in our study, NEO levels increase with age within adults, placing our results alongside findings from numerous studies investigating the link between NEO levels and aging^44^,^59^,^71^,^72^,^73^,^74^. The effect of aging on NEO in nonhuman primates has not been intensively studied, but the overall processes leading to immune system alterations with age seem to be similar to those in humans^47^,^48^. IFNγ, which is functionally closely linked to NEO and induces NEO release^28^,^29^, rather than NEO itself, is usually measured in laboratory nonhuman primate studies. Despite some inconsistencies the majority of those studies report an increase of IFNγ with older age^50^,^51^, consistent with our finding of elevated uNEO levels in aged individuals. Urinary NEO levels in our study were not linked to variation in energy balance because levels of uCP did not explain variation in uNEO levels over and above the effect of age. This suggests that increased uNEO levels in older individuals are not mediated by potential differences in nutritional status^59^,^60^ confounded with age.

Age effect size in this study was small, with a change of 4% in ten years in log-transformed uNEO levels. Elevated NEO levels are usually reported in adolescents and elderly, but not throughout adult life^75^,^76^, so we tested for a non-linear effect of aging. This was not significant, making linear modelling the better approach. Apart from the possibility that there are interspecific differences in the onset of increasing NEO levels with age between humans and macaques, this result may be driven by the lack of subadult individuals in our sample. Future studies will help to shed a light on the impact of age on uNEO in nonhuman primates. However, setting our results in context with previous results from humans including healthy adults, our results are comparable with respect to the small effect in adults^59^,^72^,^73^. Comparing the mean values of the youngest (5–7 years) and oldest (>20 years) individuals in our study group, log-transformed uNEO levels increased by 10%, which is comparable to a human study that reported a 20% increase in participants from ages 20 to 69 years^73^.

One of the major challenges in studying immunosenescence is to disentangle changes due to older age from changes due to underlying age-related disease^45^,^74^. One alternative or additional explanation for small effect size of age in our study could be the presence of many “healthy agers” more resilient to immunosenescence in our study population, since individuals more susceptible to frailty and disease are less likely to survive to old age. For this reason, very old individuals were excluded in previous studies of immunosenescence in nonhuman primates^50^. Human cross-sectional studies have demonstrated that, although increases in NEO levels are a highly consistent pattern in healthy aged individuals, this development is aggravated when combined with age related disease or overall worse health: In human gerontological studies, high NEO or IFNγ have been repeatedly linked to increased morbidity (e.g. the occurrence of rheumatoid arthritis^77^), frailty^71^ and cognitive decline^78^. In one longitudinal study on elderly people, the probability of surviving until the end of the study was linked to NEO levels, with higher NEO levels relating to lower survival probability^79^. Integrating NEO measurements into long-term projects and investigating the link between NEO levels, age and survival retrospectively will shed further light on whether this finding holds true in nonhuman primates.

Considering our findings, we conclude that uNEO has considerable potential as a non-invasive marker of immunosenescence in nonhuman primates. In general, immunosenescence mainly causes changes in T-cell subpopulations^36^,^39^, changes in monocyte or natural killer cell populations^41^,^42^, chronic, low level inflammation^43^ and overall poorer immune system performance in response to challenges, e.g. vaccination^37^,^80^, none of which can be investigated non-invasively to date. However, these immune system changes correlate with increased NEO levels^41^. Additionally, adverse health factors, such as HIV infection^40^, inflammatory processes and prolonged physiological stress^36^,^43^ have been shown to induce changes in the immune system that mirror immunosenescence. These are assumed to accelerate and intensify age related changes in the immune system while being linked to elevated NEO levels^40^,^41^. Thus, uNEO could serve as an immunosenescence marker, as well as a marker for overall health in wildlife, with chronically elevated uNEO levels indicative of worse health and lower expected survival time in aged individuals. Despite intensive efforts to define consistent changes throughout life history in humans^74^,^75^,^76^, defining markers and threshold values for healthy aging remains challenging. NEO offers a valuable addition to studies of health and aging in wildlife, particularly if combined with other health-linked parameters, such as glucocorticoid hormone levels, visual frailty, wound healing patterns, or energy status, when addressing question of eco-immunology, health and fitness under natural conditions.

The picture concerning the impact of parasites on uNEO levels is less clear, as we found no changes of uNEO levels in response to anthelmintic treatment. Based on the general pattern of immune responses against GI parasites and previous findings in buffaloes^24^,^54^, where parasite clearance increased Th1-activity in treated individuals^24^,^54^, we predicted an increase in uNEO levels in response to anthelmintic treatment. Since NEO is connected to Th1 responses^28^,^81^, inflammation^28^,^71^, acute stress^32^,^71^ and severity of various infectious and non-infectious diseases in humans^28^, the lack of evidence for an impact of parasite removal on uNEO levels in this study is surprising. However, several factors could lead to the effects of treatment being masked or attenuated in our study: First, the anthelmintic compound used for treatment, ivermectin, has been reported to improve immune function in studies on various species, e.g. humans, sheep, pigs, rabbits and rats^82^,^83^,^84^,^85^. However most studies used long-term treatment rather than single doses^82^, higher than therapeutic dosages^83^, ivermectin treatment in absence of parasites, or experimental infections with a single parasite species^85^,^86^ and reported only transient effects of ivermectin^83^. Therefore, we conclude that the impact of the ivermectin treatment on uNEO levels in our study is probably negligible.

Second, different parasite species elicit distinct and highly specific immune responses. Both hookworms and *Oesophagostomum* spp. have been demonstrated to elicit mixed Th1/Th2 type responses^87^ and *Oesophagostomum* spp. tend to cause chronic rather than transient infections^88^, potentially due to a Th1-directed shift and lack of protective immunity^87^. Likewise, *Trichuris* infections are usually transient^88^, but can become chronic if animals fail to mount efficient Th2-responses^89^. These shifts towards a more Th1 prone response are not only based on host genetics^24^,^89^, but can also be induced by the parasites themselves in an arms-race with their host. Evidence is mounting that GI parasites actively manipulate the host immune system via excretory substances^90^, e.g. both human hookworms and murine *Trichuris* produce substances that mimic IFNγ or induce its release^91^,^92^. Consequently, both immunomodulation by the parasites and parasite clearance can cause heightened Th1 responses^24^,^92^, leading to unchanged uNEO levels after treatment.

Third, several individuals in our study became GI parasite positive again quickly after treatment, as indicated by shedding of parasite eggs within four weeks after treatment. This could explain the difference between our lack of finding and the results of studies on ungulates where individuals treated with a compound bolus were parasite free for up to 24 months^24^,^54^. We can expect tissue damage due to either wandering hookworm larvae^91^,^93^ or encystation of *Oesophagostomum* larvae in the gut mucosa^63^ in freshly infected individuals. Early immune responses are expected against parasitic antigens as well as tissue damage. Consequently, it is possible that changes in uNEO levels occur on a short timescale which we were unable to capture with our sampling regime. Similarly, the effects of SIV infection in experimentally infected rhesus and longtailed macaques were pronounced, but transient, with clearly elevated uNEO levels between 10–20 days after infection and subsequently moderately elevated levels^26^, with the changes expected in our experiment being smaller than those in an infection with SIV.

Fourth and most importantly, individuals in the study population are treated regularly and are thus infected repeatedly on a regular basis. Laboratory studies on immune-parasite interactions are often on first infections, which usually elicit strong immune responses^94^. Subsequent infections often lead to less pronounced immune response patterns^95^,^96^. Even in infections such as malaria in which NEO levels are expected to rise markedly, repeated infections have been demonstrated to attenuate the NEO response^97^. Given that for most host individuals, parasite burdens will be low while only a few show high parasite burdens^98^ and natural infections with GI parasites usually occur via low-dose trickle infections^96^, changes in the infection status may not necessarily lead to pronounced changes in the immune system balance of most hosts. Additionally, infections early in life, which are to be expected in our study population, have been shown to induce long lasting shifts of the immune system towards Th2 responses that are not influenced by repeated anthelmintic treatment^99^, a scenario that might well mimic the situation of our study population. Further studies using more frequent sampling and focussing on first reinfections in young individuals might help to evaluate whether NEO can be used as an indirect marker of GI parasites in nonhuman primates.

## Conclusion

In accordance with previous studies on human and nonhuman primates, aged Barbary macaques of our study groups showed higher uNEO levels compared to younger adult individuals. This leads us to conclude that semi-free ranging Barbary macaques, presumably faced with a more natural range of pathogens than are laboratory nonhuman primates, show signs of immunosenescence which can be measured by uNEO levels. For these reasons, we argue that uNEO is a potential marker for studies on wildlife health, particularly for studies on the effects of aging, despite the lack of effect of parasite clearance on uNEO in our study subjects. We suggest future studies linking longitudinal data on uNEO levels with data on individuals’ survival and reproductive success. Including uNEO measurements might prove a valuable contribution to studies of aging, decreased immune function, morbidity and factors determining longevity and survival in wildlife.

## Additional Information

**How to cite this article:** Müller, N. *et al*. Age, but not anthelmintic treatment, is associated with urinary neopterin levels in semi-free ranging Barbary macaques. *Sci. Rep.* **7**, 41973; doi: 10.1038/srep41973 (2017).

**Publisher's note:** Springer Nature remains neutral with regard to jurisdictional claims in published maps and institutional affiliations.

## Figures and Tables

**Table 1:** Overview over sampled individuals and samples used for uNEO analysis.

social*experimental group	H control		H treatment		C treatment	
sex	female	male	female	male	female	male
nr. individuals						
young (5–7 years)	3	2	2	1	2	1
prime (8–14 years)	3	3	4	3	1	1
post prime (15–19 years)	2	1	1	1	—	—
aged (>20 years)	1	3	3	3	—	2
sum individuals = 43						
nr. samples (mean ± sd /individual)						
pre	19 (2.1 ± 0.9)	21 (2.3 ± 0.7)	22 (2.2 ± 0.8)	20 (2.5 ± 0.8)	5 (1.7 ± 0.6)	6 (1.5 ± 0.6)
post	21 (2.3 ± 0.7)	20 (2.2 ± 0.8)	19 (1.9 ± 0.9)	18 (2.3 ± 0.9)	3 (1 ± 0)	5 (1.3 ± 0.5)
sum samples = 179 (4.2 ± 1.3)						

Distribution of study animals among social groups, age and sex classes as well as sample sizes are given for treatment and control group. Age classes were chosen for means of better visualization of data distribution, but were not used in data analyses or interpretation.

**Table 2:** uNEO levels (ng/ml corr. SG) in 43 adult semi-free ranging Barbary macaques at *Affenberg Salem*.

all samples	mean ± sd range	160.45 ± 90.50 30.68–572.73	
by phase * exp. group	control	treatment	
pre	164.24 ± 65.21 (54.55–322.89)	170.80 ± 110.39 (59.09–518.18)	
post	157.32 ± 70.10 (43.64–386.36)	148.03 ± 100.69 (30.68–572.73)	
by age class			
young (5–7 years)	123.75 ± 56.11 (30.68–295.80)	**post prime (15–19 years)**	166.38 ± 88.63 (40.91–386.36)
prime (8–14 years)	151.29 ± 81.51 (43.64–454.91	**aged (>20 years)**	200.88 ± 109.77 (71.59–572.73)

For each category, mean ± standard deviation and range (min to max) are given. Age classes were chosen for means of better visualization of data distribution, but were not used in data analyses or interpretation.

**Table 3:** Results of LMM (Model 1, n = 179 samples) for the effects of age and the three-way interaction of experimental group, phase and *Capillaria* infection (*Cap*) on uNEO levels.

	estimate	std.error	confidence intervals		df	t-value	p-value
			**2.5%**	**97.5%**			
**intercept**	4.65	0.27	4.06	5.22	1.79	17.09	0.005
**age**	**0.02**	**0.01**	**0.01**	**0.03**	**34.92**	**2.58**	0.014 *
cap*exp.group*phase	0.38	0.27	−0.13	0.90	139.57	1.44	0.15
cap*exp.group	0.09	0.24	−0.35	0.55	71.88	0.40	0.69
cap*phase	−0.10	0.19	−0.47	0.27	141.28	−0.51	0.62
exp.group*phase	−0.03	0.17	−0.37	0.30	145.43	−0.21	0.83
**exp.group**	−0.09	0.15	−0.39	0.20	70.67	−0.55	0.58
**phase**	0.09	0.14	−0.18	0.35	18.10	0.62	0.54
**cap**	0.04	0.18	−0.30	0.38	62.09	0.22	0.82
**sex**	0.10	0.10	−0.08	0.29	37.00	1.08	0.29
**log(uCP (ng/ml corr. SG))**	0.03	0.02	−0.01	0.08	165.46	1.35	0.18

The two-way interactions between *Capillaria*, experimental group and phase were retained although uninterpretable. The single terms experimental group, phase and *Cap*, sex and uCP levels were included as control variables, social group and individual ID as random factors.

**Table 4:** Results of LMM (Model 2, n = 179 samples) for the effects of age and the two-way interaction of experimental group and phase on uNEO levels.

	estimate	std.error	confidence intervals		df	t-value	p-value
			2.5%	97.5%			
**intercept**	4.603	0.270	4.003	5.181	1.580	17.050	0.009
**age**	**0.019**	**0.007**	**0.004**	**0.034**	**36.320**	**2.452**	0.019 *
exp.group*phase	0.110	0.130	−0.144	0.364	142.520	0.846	0.399
**exp.group**	−0.039	0.122	−0.283	0.185	66.880	−0.381	0.752
**phase**	0.036	0.097	−0.152	0.224	143.410	0.369	0.713
**cap**	0.152	0.115	−0.064	0.372	39.650	1.324	0.193
**sex**	0.083	0.100	−0.109	0.227	37.400	0.827	0.413
**log(uCP (ng/ml corr. SG))**	0.033	0.023	−0.011	0.078	168.380	1.443	0.151

Experimental group, phase, *Capillaria* infection (*Cap*), sex, and uCP levels were included as control variables, social group and individual ID as random factors.

## References

[b1] Nunn C. L. & Altizer S. Infectious Diseases in Primates: Behavior, Ecology and Evolution (Oxford Series in Ecology and Evolution. Oxford University Press, 2006).

[b2] Tompkins D. M., Dunn A. M., Smith M. J. & Telfer S. Wildlife diseases: from individuals to ecosystems. Journal of Animal Ecology 80, 19–38 (2011). 20735792 10.1111/j.1365-2656.2010.01742.x

[b3] Freeland W. Pathogens and the evolution of primate sociality. Biotropica 8, 12–24 (1976).

[b4] Hill A. V. The immunogenetics of human infectious diseases. Annual Review of Immunology 16, 593–617 (1998). 10.1146/annurev.immunol.16.1.593 9597143

[b5] Karlsson E. K., Kwiatkowski D. P. & Sabeti P. C. Natural selection and infectious disease in human populations. Nature Review Genetics 15, 379–393 (2014). 10.1038/nrg3734 PMC4912034 24776769

[b6] Buer J. & Balling R. Mice, microbes and models of infection. Nature Review Genetics 4, 195–205 (2003). 10.1038/nrg1019 12610524

[b7] Gardner M. B. & Luciw P. A. Macaque models of human infectious disease. ILAR journal/National Research Council, Institute of Laboratory Animal Resources 49, doi: 10.1093/ilar.49.2.220 (2008). PMC7108592 18323583

[b8] Altizer S. . Social organization and parasite risk in mammals: integrating theory and empirical studies. Annual Review of Ecology and Systematics 34, 517–547 (2003).

[b9] Nussey D. H., Froy H., Lemaitre J.-F., Gaillard J.-M. & Austad S. N. Senescence in natural populations of animals: Widespread evidence and its implications for bio-gerontology. Ageing Research Reviews 12, 214–225 (2013). 22884974 10.1016/j.arr.2012.07.004 PMC4246505

[b10] Watson R. L. . Cellular and humoral immunity in a wild mammal: Variation with age & sex and association with overwinter survival. Ecology and Evolution, doi: 10.1002/ece3.2584 (2016). PMC5192870 28035261

[b11] Jégo M. . Haematological parameters do senesce in the wild: evidence from different populations of a long-lived mammal. Journal of Evolutionary Biology 27, 2745–2752 (2014). 25358546 10.1111/jeb.12535

[b12] MacIntosh A. J. J. . Monkeys in the middle: Parasite transmission through the social network of a wild primate. PLoS ONE 7, doi: 10.1371/journal.pone.0051144 (2012). PMC3515516 23227246

[b13] Young C., Majolo B., Heistermann M., Schülke O. & Ostner J. Responses to social and environmental stress are attenuated by strong male bonds in wild macaques. Proceedings of the National Academy of Sciences of the United States of America 111, 18195–18200 (2014). 25489097 10.1073/pnas.1411450111 PMC4280642

[b14] Muehlenbein M. & Bribiescas R. Testosterone-mediated immune functions and male life histories. American Journal of Human Biology 17, 527–558 (2005). 16136532 10.1002/ajhb.20419

[b15] Muehlenbein M. P. & Watts D. P. The costs of dominance: testosterone, cortisol and intestinal parasites in wild male chimpanzees. Biopsychosocial Medicine 4, 21–21 (2010). 21143892 10.1186/1751-0759-4-21 PMC3004803

[b16] Maréchal L., Semple S., Majolo B. & MacLarnon A. Assessing the effects of tourist provisioning on the health of wild Barbary macaques in Morocco. PLoS ONE 11, doi: 10.1371/journal.pone.0155920 (2016). PMC4874683 27203861

[b17] Borg C., Majolo B., Qarro M. & Semple S. A Comparison of body size, coat condition and endoparasite diversity of wild Barbary macaques exposed to different levels of tourism. Anthrozoos 27, 49–63 (2014).

[b18] Archie E. A., Altmann J. & Alberts S. C. Social status predicts wound healing in wild baboons. Proceedings of the National Academy of Sciences 109, 9017–9022 (2012). 10.1073/pnas.1206391109 PMC3384186 22615389

[b19] Grueter C. C., Deschner T., Behringer V., Fawcett K. & Robbins M. M. Socioecological correlates of energy balance using urinary C-peptide measurements in wild female mountain gorillas. Physiology & Behavior 127, 13–19 (2014). 24472322 10.1016/j.physbeh.2014.01.009

[b20] Girard-Buttoz C. . Urinary C-peptide measurement as a marker of nutritional status in macaques. PLoS ONE 6, doi: 10.1371/journal.pone.0018042 (2011). PMC3068145 21479215

[b21] Cristóbal-Azkarate J., Maréchal L., Semple S., Majolo B. & MacLarnon A. Metabolic strategies in wild male Barbary macaques: evidence from faecal measurement of thyroid hormone. Biology Letters 12, doi: 10.1098/rsbl.2016.0168 (2016). PMC4881361 27095269

[b22] Schaebs F. S., Wolf T. E., Behringer V. & Deschner T. Fecal thyroid hormones allow for the noninvasive monitoring of energy intake in capuchin monkeys. Journal of Endocrinology 231, 1–10 (2016). 27460343 10.1530/JOE-16-0152

[b23] Graham A. L. . Fitness Correlates of heritable variation in antibody responsiveness in a wild mammal. Science 330, 662–665 (2010). 21030656 10.1126/science.1194878

[b24] Ezenwa V. O., Etienne R. S., Luikart G., Beja-Pereira A. & Jolles A. E. Hidden consequences of living in a wormy world: Nematode-induced immune suppression facilitates Tuberculosis invasion in African buffalo. The American Naturalist 176, 613–624 (2010). 10.1086/656496 20849271

[b25] Abbott D. H. . Are subordinates always stressed? A comparative analysis of rank differences in cortisol levels among primates. Hormones and Behavior 43, 67–82 (2003). 12614636 10.1016/s0018-506x(02)00037-5

[b26] Higham J. P. . Evaluating noninvasive markers of nonhuman primate immune activation and inflammation. American Journal of Physical Anthropology 158, 673–684 (2015). 26250063 10.1002/ajpa.22821

[b27] Heistermann M. & Higham J. P. Urinary neopterin, a non-invasive marker of mammalian cellular immune activation, is highly stable under field conditions. Scientific Reports 5, 16308, doi: 10.1038/srep16308 (2015). 26549509 PMC4637859

[b28] Murr C., Widner B., Wirleitner B. & Fuchs D. Neopterin as a marker for immune system activation. Current Drug Metabolism 3, 175–187 (2002). 12003349 10.2174/1389200024605082

[b29] Widner B. . The importance of neopterin as a laboratory diagnostic marker of immune activation. Pteridines 10, 101–111 (1999).

[b30] Husain N. . Neopterin concentration as an index of disease activity in Crohn’s disease and ulcerative colitis. Journal of Clinical Gastroenterology 47, 246–251 (2013). 23269308 10.1097/MCG.0b013e3182582cdb

[b31] Unal B. . Serum neopterin as a prognostic indicator in patients with gastric carcinoma. Journal of Investigative Surgery 22, 419–425 (2009). 20001811 10.3109/08941930903410783

[b32] Breineková K., Svoboda M., Smutná M. & Vorlová L. Markers of acute stress in pigs. Physiological Research. 56, 323–329 (2007). 16792472 10.33549/physiolres.930938

[b33] Berdowska A. & Zwirska-Korczala K. Neopterin measurement in clinical diagnosis. Journal of Clinical Pharmacy and Therapeutics 26, 319–329 (2001). 11679022 10.1046/j.1365-2710.2001.00358.x

[b34] Aulitzky W. E. . Comparison of serum neopterin levels and urinary neopterin excretion in renal allograft recipients. Clinical Nephrology 29, 248–252 (1988). 3293855

[b35] Fendrich C. . Urinary neopterin concentrations in rhesus monkeys after infection with simian immunodeficiency virus (SIVmac 251). AIDS 3, 305–308 (1989). 2548536 10.1097/00002030-198905000-00010

[b36] Hawkley L. C. & Cacioppo J. T. Stress and the aging immune system. Brain, Behavior, and Immunity 18, 114–119 (2004). 10.1016/j.bbi.2003.09.005 14986706

[b37] Goodwin K., Viboud C. & Simonsen L. Antibody response to influenza vaccination in the elderly: a quantitative review. Vaccine 24, doi: 10.1016/j.vaccine.2005.08.105 (2006). 16213065

[b38] Li H., Manwani B. & Leng S. X. Frailty, inflammation, and immunity. Aging and Disease 2, 466–473 (2014). PMC3295062 22396895

[b39] Faria A. M. C. . Variation rhythms of lymphocyte subsets during healthy aging. Neuroimmunomodulation 15, 365–379 (2008). 19047812 10.1159/000156478

[b40] Deeks S. G. HIV infection, inflammation, immunosenescence, and aging. Annual Review of Medicine 62, 141–155 (2011). 10.1146/annurev-med-042909-093756 PMC3759035 21090961

[b41] Hearps A. C. . Aging is associated with chronic innate immune activation and dysregulation of monocyte phenotype and function. Aging Cell 11, 867–875 (2012). 22708967 10.1111/j.1474-9726.2012.00851.x

[b42] Solana R. . Innate immunosenescence: Effect of aging on cells and receptors of the innate immune system in humans. Seminars in Immunology 24, 331–341 (2012). 22560929 10.1016/j.smim.2012.04.008

[b43] Bauer M. E., Jeckel C. M. M. & Luz C. The role of stress factors during aging of the immune system. Annals of the New York Academy of Sciences 1153, 139–152 (2009). 19236337 10.1111/j.1749-6632.2008.03966.x

[b44] Frick B., Schroecksnadel K., Neurauter G., Leblhuber F. & Fuchs D. Increasing production of homocysteine and neopterin and degradation of tryptophan with older age. Clinical Biochemistry 37, 684–687 (2004). 15302611 10.1016/j.clinbiochem.2004.02.007

[b45] Murr C. . Increased neopterin concentration in older age coincides with decline of CD28 + CD45RA + T-cells. Pteridines 15, 170–174 (2004).

[b46] Anderson R. M. & Colman R. J. Prospects and perspectives in primate aging research. Antioxidants & Redox Signaling 14, 203–205 (2011). 20712396 10.1089/ars.2010.3227 PMC3000241

[b47] Messaoudi I., Estep R., Robinson B. & Wong S. W. Nonhuman primate models of human immunology. Antioxidants & Redox Signaling 14, 261–273 (2011). 20524846 10.1089/ars.2010.3241 PMC3014769

[b48] Meyer C., Kerns A., Haberthur K. & Messaoudi I. Improving immunity in the elderly: current and future lessons from nonhuman primate models. Age 34, 1157–1168 (2012). 22180097 10.1007/s11357-011-9353-y PMC3448983

[b49] Weng N.-P. Aging of the immune system: How much can the adaptive immune system adapt? Immunity 24, 495–499 (2006). 16713964 10.1016/j.immuni.2006.05.001 PMC2266981

[b50] Didier E. S., Sugimoto C., Bowers L. C., Khan I. A. & Kuroda M. J. Immune correlates of aging in outdoor-housed captive rhesus macaques (*Macaca mulatta*). Immunity & Ageing 9, 1–15 (2012). 23151307 10.1186/1742-4933-9-25 PMC3541156

[b51] Haberthur K., Engelman F., Barron A. & Messaoudi I. Immune senescence in aged nonhuman primates. Experimental Gerontology 45, doi: 10.1016/j.exger.2010.06.001 (2010). PMC2926233 20558288

[b52] Huffman M. A. & Chapman C. A. Primate Parasite Ecology: The Dynamics and Study of Host-Parasite relationships (ed Cambridge University Press) (Cambridge, 2009).

[b53] Ferrari N., Cattadori I. M., Nespereira J., Rizzoli A. & Hudson P. J. The role of host sex in parasite dynamics: field experiments on the yellow-necked mouse Apodemus flavicollis. Ecology Letters 7, 88–94 (2004).

[b54] Ezenwa V. O. & Jolles A. E. Opposite effects of anthelmintic treatment on microbial infection at individual versus population scales. Science 347, 175–177 (2015). 25574023 10.1126/science.1261714

[b55] Long K. Z. & Nanthakumar N. Energetic and nutritional regulation of the adaptive immune response and trade-offs in ecological immunology. American Journal of Human Biology 16, 499–507 (2004). 15368598 10.1002/ajhb.20064

[b56] Carvalho L. . Review series on helminths, immune modulation and the hygiene hypothesis: Mechanisms underlying helminth modulation of dendritic cell function. Immunology 126, 28–34 (2009). 19120496 10.1111/j.1365-2567.2008.03008.x PMC2632707

[b57] Derting Terry L. & Compton S. Immune response, not immune maintenance, is energetically costly in wild white-footed mice (*Peromyscus leucopus*). Physiological and Biochemical Zoology 76, 744–752 (2003). 14671721 10.1086/375662

[b58] Martin L. B., Scheuerlein A. & Wikelski M. Immune activity elevates energy expenditure of house sparrows: a link between direct and indirect costs? *Proceedings of the Royal Society of London*. Series B: Biological Sciences 270, 153–158 (2003). 10.1098/rspb.2002.2185 PMC1691219 12590753

[b59] Spencer M. E. . Serum levels of the immune activation marker neopterin change with age and gender and are modified by race, BMI, and percentage of body fat. The Journals of Gerontology Series A: Biological Sciences and Medical Sciences 65A, 858–865 (2010). 10.1093/gerona/glq066 PMC2903784 20478905

[b60] Ledochowski M., Murr C., Widner B. & Fuchs D. Association between insulin resistance, body mass and neopterin concentrations. Clinica Chimica Acta 282, 115–123 (1999). 10.1016/s0009-8981(99)00019-4 10340439

[b61] Deschner T., Kratzsch J. & Hohmann G. Urinary C-peptide as a method for monitoring body mass changes in captive bonobos (*Pan paniscus*). Hormones and Behavior 54, 620–626 (2008). 18638479 10.1016/j.yhbeh.2008.06.005

[b62] de Turckheim G. & Merz E. in The Barbary macaque: a case study in conservation (ed Fa J. E.) 241–261 (Plenum Press, 1984).

[b63] Dash K. M. The life cycle of *Oesophagostomum columbianum* (Curtice, 1890) in sheep. International Journal for Parasitology 3, 843–851 (1973). 4762128 10.1016/0020-7519(73)90075-1

[b64] Danish L. M., Heistermann M., Agil M. & Engelhardt A. Validation of a novel collection device for non-invasive urine sampling from free-ranging animals. PLoS ONE 10, doi: 10.1371/journal.pone.0142051 (2015). PMC4633224 26536024

[b65] Higham J. P., Girard-Buttoz C. d., Engelhardt A. & Heistermann M. Urinary C-peptide of insulin as a non-invasive marker of nutritional status: some practicalities. PLoS ONE 6, doi: 10.137/journal.pone.0022398 (2011). PMC3142156 21799844

[b66] Gillespie T. R. Noninvasive assessment of gastrointestinal parasite infections in free-ranging primates. International Journal of Primatology 27, 1129–1143 (2006).

[b67] Chandra R. K. Nutrition and the immune system: an introduction. The American Journal of Clinical Nutrition 66, 460S–463S (1997). 9250133 10.1093/ajcn/66.2.460S

[b68] Emery Thompson M., Muller M. N. & Wrangham R. W. Technical note: Variation in muscle mass in wild chimpanzees: Application of a modified urinary creatinine method. American Journal of Physical Anthropology 149, 622–627 (2012). 23077085 10.1002/ajpa.22157

[b69] Miller R. C. . Comparison of specific gravity and creatinine for normalizing urinary reproductive hormone concentrations. Clinical Chemistry 50, 924–932 (2004). 15105350 10.1373/clinchem.2004.032292

[b70] R development core team. A language and environment for statistical computing. (R foundation for statistical computing, 2011).

[b71] Capuron L. . Activated immune system and inflammation in healthy ageing: Relevance for tryptophan and neopterin metabolism. Current Pharmaceutical Design 20, 6048–6057 (2014). 24641220 10.2174/1381612820666140317110217

[b72] Schennach H. . Factors influencing serum neopterin concentrations in a population of blood donors. Clinical Chemistry 48, 643–645 (2002). 11901063

[b73] Diamondstone L. S. . Factors influencing serum neopterin and ß2-microglobulin levels in a healthy diverse population. Journal of Clinical Immunology 14, 368–374 (1994). 7883864 10.1007/BF01546321

[b74] Reibnegger G. . Approach to define “normal aging” in man. Immune function, serum lipids, lipoproteins and neopterin levels. Mechanisms of Ageing and Development 46, 67–82 (1988). 3226163 10.1016/0047-6374(88)90115-7

[b75] Werner E. R. . Determination of neopterin in serum and urine. Clinical Chemistry 33, 62–66 (1987). 3802497

[b76] Shintaku H., Isshiki G., Hase Y., Tsuruhara T. & Oura T. Normal pterin values in urine and serum in neonates and its age-related change throughout life. Journal of Inherited Metabolic Disease 5, 241–242 (1982). 6820449 10.1007/BF02179155

[b77] Arshadi D. . Plasma level of neopterin as a marker of disease activity in treated rheumatoid arthritis patients: Association with gender, disease activity and anti-CCP antibody. International Immunopharmacology 17, 763–767 (2013). 24055018 10.1016/j.intimp.2013.08.022

[b78] Parker D. C. . Plasma neopterin level as a marker of peripheral immune activation in amnestic mild cognitive impairment and Alzheimer’s disease. International Journal of Geriatric Psychiatry 28, 149–154 (2013). 22539447 10.1002/gps.3802 PMC3505262

[b79] Solichová D., Melichar B., Svobodová I., Bláha V. & Zadák Z. Fluorescence analysis of antioxidant vitamins and neopterin in nonagenarians. Biomedical Chromatography, 117–118 (1999).

[b80] Čičin-Šain L. . Loss of naive T-cells and repertoire constriction predict poor response to vaccination in old primates. Journal of immunology (Baltimore, Md.: 1950) 184, 6739–6745 (2010). 10.4049/jimmunol.0904193 PMC3504654 20483749

[b81] Elenkov I. J. & Chrousos G. P. Stress hormones, Th1/Th2 patterns, pro/anti-inflammatory cytokines and susceptibility to disease. Trends in Endocrinology and Metabolism 10, 359–368 (1999). 10511695 10.1016/s1043-2760(99)00188-5

[b82] Steel C., Lujan-Trangay A., Gonzalez-Peralta C., Zea-Flores G. & Nutman T. B. Transient changes in cytokine profiles following ivermectin treatment of onchocerciasis. Journal of Infectious Diseases 170, 962–970 (1994). 7930742 10.1093/infdis/170.4.962

[b83] Sajid M. S. . Effect of ivermectin on the cellular and humoral immune responses of rabbits. Life Sciences 80, 1966–1970 (2007). 17379254 10.1016/j.lfs.2007.02.025

[b84] López-Olvera J. R., Höfle U., Vicente J., Fernández-de-Mera I. G. & Gortázar C. Effects of parasitic helminths and ivermectin treatment on clinical parameters in the European wild boar (Sus scrofa). Parasitology Research 98, 582–587 (2006). 16437240 10.1007/s00436-005-0099-2

[b85] Stankiewicz M. . Influence of ivermectin on cellular and humoral immune responses of lambs. Veterinary Immunology and Immunopathology 44, 347–358 (1995). 7747411 10.1016/0165-2427(94)05308-f

[b86] Uhlíř J. & Volf P. Ivermectin: its effect on the immune system of rabbits and rats infested with ectoparasites. Veterinary Immunology and Immunopathology 34, 325–336 (1992). 1455687 10.1016/0165-2427(92)90173-n

[b87] Pit D., Polderman A., Baeta S., Schulz-Key H. & Soboslay P. Parasite-specific antibody and cellular immune responses in humans infected with *Necator americanus* and *Oesophagostomum bifurcum*. Parasitology Research 87, 722–729 (2001). 11570557 10.1007/s004360100419

[b88] Andreasen A. . Immune and inflammatory responses in pigs infected with *Trichuris suis* and *Oesophagostomum dentatum*. Veterinary Parasitology 207, 249–258 (2015). 25576439 10.1016/j.vetpar.2014.12.005

[b89] Else K. J. & Grencis R. K. Cellular immune-responses to the murine nematode parasite *Trichuris-muris*.1. Differential cytokine production during acute or chronic infection. Immunology 72, 508–513 (1991). 1903765 PMC1384369

[b90] Hewitson J. P., Grainger J. R. & Maizels R. M. Helminth immunoregulation: The role of parasite secreted proteins in modulating host immunity. Molecular and Biochemical Parasitology 167, 1–11 (2009). 19406170 10.1016/j.molbiopara.2009.04.008 PMC2706953

[b91] Hsieh G. C.-F. . A secreted protein from the human hookworm *Necator americanus* binds selectively to NK cells and induces IFN-γ production. The Journal of Immunology 173, 2699–2704 (2004). 15294988 10.4049/jimmunol.173.4.2699

[b92] Grencis R. K. & Entwistle G. M. Production of an interferon-gamma homologue by an intestinal nematode: functionally significant or interesting artefact? Parasitology 115, 101–105 (1997). 9571695 10.1017/s0031182097002114

[b93] Hotez P. J. . Hookworm infection. New England Journal of Medicine 351, 799–807 (2004). 15317893 10.1056/NEJMra032492

[b94] Reber L. L., Sibilano R., Mukai K. & Galli S. J. Potential effector and immunoregulatory functions of mast cells in mucosal immunity. Mucosal immunology 8, 444–463 (2015). 25669149 10.1038/mi.2014.131 PMC4739802

[b95] Maizels R. M. & Yazdanbakhsh M. Immune regulation by helminth parasites: cellular and molecular mechanisms. Nature Reviews Immunology 3, 733–744 (2003). 10.1038/nri1183 12949497

[b96] Grencis R. K., Humphreys N. E. & Bancroft A. J. Immunity to gastrointestinal nematodes: mechanisms and myths. Immunological Reviews 260, 183–205 (2014). 24942690 10.1111/imr.12188 PMC4141702

[b97] Brown A. E., Webster H. K., Teja-Isavadharm P. & Keeratithakul D. Macrophage activation in falciparum malaria as measured by neopterin and interferon-gamma. Clinical & Experimental Immunology 82, 97–101 (1990). 2119922 10.1111/j.1365-2249.1990.tb05410.x PMC1535156

[b98] Hawley D. M. & Altizer S. M. Disease ecology meets ecological immunology: understanding the links between organismal immunity and infection dynamics in natural populations. Functional Ecology 25, 48–60 (2011).

[b99] Wright V. J. . Early exposure of infants to GI nematodes induces Th2 dominant immune responses which are unaffected by periodic anthelminthic treatment. PLoS Neglected Tropical Diseases 3, doi: 10.1371/journal.pntd.0000433 (2009). PMC2677666 19436745

